# Supplementary material for: Efficient Isolation and Functional Characterization of Niche Cells from Human Corneal Limbus
Source: Int J Mol Sci. 2022 Mar 2;23(5):2750. doi: 10.3390/ijms23052750 (PMC8911296; doi:10.3390/ijms23052750)
Supplement: Supplementary file 1 [file ijms-23-02750-s001.zip › ijms-1611815-supplementary.pdf]

**Efficient isolation and functional characterisation of niche cells from human corneal limbus**

Naresh Polisetti[1], Lyne Sharaf[1], Ursula Schlötzer-Schrehardt[2], Günther Schlunck[1], Thomas Reinhard[1]

Supplementary Table S1: Organ cultured corneal scleral tissues used in this study.

| S.no. | Age (Years) | Post mortem time (h) | Duration of cultivation (days) |
|---|---|---|---|
| 1 | 78 | 12.5 | 27 |
| 2 | 63 | 42.1 | 28 |
| 3 | 68 | 17.9 | 28 |
| 4 | 68 | 17.9 | 30 |
| 5 | 59 | 30.1 | 27 |
| 6 | 80 | 39.5 | 27 |
| 7 | 71 | 64.3 | 20 |
| 8 | 71 | 64.3 | 22 |
| 9 | 67 | 27.4 | 19 |
| 10 | 67 | 27.4 | 22 |
| 11 | 74 | 23.5 | 26 |
| 12 | 75 | 38.3 | 20 |
| 13 | 78 | 60.3 | 23 |
| 14 | 78 | 60.3 | 23 |
| 15 | 74 | 32.1 | 23 |
| 16 | 74 | 32.1 | 20 |
| 17 | 68 | 28.5 | 21 |
| 18 | 84 | 20.2 | 26 |
| 19 | 58 | 58.4 | 23 |
| 20 | 58 | 58.4 | 21 |
| 21 | 55 | 30.1 | 29 |
| 22 | 60 | 24.1 | 15 |
| 23 | 60 | 24.1 | 20 |
| 24 | 65 | 23.3 | 19 |
| 25 | 65 | 23.3 | 19 |
| 26 | 72 | 11.0 | 18 |
| 27 | 72 | 11.0 | 27 |
| 28 | 55 | 26.5 | 25 |
| 29 | 88 | 15.2 | 19 |
| 30 | 88 | 15.2 | 24 |
| 31 | 83 | 65.4 | 23 |
| 32 | 83 | 65.4 | 21 |
| 33 | 56 | 41.5 | 23 |
| 34 | 56 | 41.5 | 21 |
| 35 | 86 | 64.0 | 29 |
| 36 | 82 | 16.4 | 21 |
| 37 | 64 | 30.4 | 25 |
| 38 | 74 | 63.3 | 23 |
| 39 | 75 | 18.0 | 21 |
| 40 | 83 | 14.5 | 27 |
| 41 | 83 | 14.5 | 25 |
| 42 | 56 | 26.4 | 26 |
| 43 | 56 | 26.4 | 19 |

| | | | |
|---|---|---|---|
| 44 | 54 | 68.2 | 21 |
| 45 | 54 | 68.2 | 21 |
| 46 | 84 | 19.4 | 23 |
| 47 | 84 | 19.4 | 21 |
| 48 | 64 | 28.0 | 22 |
| 49 | 64 | 28.0 | 27 |
| 50 | 59 | 33.2 | 28 |
| 51 | 59 | 33.2 | 28 |
| 52 | 72 | 27.3 | 26 |
| 53 | 71 | 47.3 | 25 |
| 54 | 79 | 26.4 | 26 |
| 55 | 79 | 26.4 | 24 |
| 56 | 71 | 29.0 | 2 |
| 57 | 71 | 29.0 | 23 |
| 58 | 63 | 26.1 | 28 |
| 59 | 63 | 26.1 | 28 |
| 60 | 87 | 18.3 | 29 |
| 61 | 80 | 68.3 | 28 |
| 62 | 80 | 68.3 | 26 |
| 63 | 72 | 36.0 | 24 |
| 64 | 72 | 36.0 | 26 |
| 65 | 64 | 22.3 | 26 |
| 66 | 64 | 22.3 | 24 |
| 67 | 67 | 42.3 | 28 |
| 68 | 67 | 42.3 | 30 |
| 69 | 70 | 47.3 | 22 |
| 70 | 70 | 47.3 | 27 |
| 71 | 74 | 19.0 | 25 |
| 72 | 74 | 19.0 | 25 |
| 73 | 70 | 35.5 | 26 |
| 74 | 74 | 18.1 | 27 |
| 75 | 74 | 18.1 | 1 |
| 76 | 53 | 45.5 | 28 |
| 77 | 61 | 59.4 | 28 |
| 78 | 67 | 11.3 | 26 |
| 79 | 67 | 11.3 | 26 |
| 80 | 85 | 42.1 | 1 |
| 81 | 76 | 24.4 | 29 |
| 82 | 48 | 18.3 | 24 |
| 83 | 79 | 21.0 | 21 |
| 84 | 80 | 69.3 | 22 |
| 85 | 70 | 15.1 | 21 |
| 86 | 70 | 15.1 | 28 |
| 87 | 84 | 65.4 | 27 |
| 88 | 84 | 65.4 | 25 |
| 89 | 78 | 24.1 | 25 |
| 90 | 78 | 24.1 | 25 |
| 91 | 68 | 25.3 | 24 |
| 92 | 68 | 25.3 | 26 |
| 93 | 89 | 24.4 | 24 |
| 94 | 31 | 69.1 | 23 |
| 95 | 68 | 44.4 | 26 |
| 96 | 63 | 41.0 | 26 |
| 97 | 77 | 41.1 | 21 |

| | | | |
|---|---|---|---|
| 98 | 63 | 36.1 | 28 |
| 99 | 54 | 43.2 | 28 |
| 100 | 54 | 43.2 | 28 |
| 101 | 77 | 43.2 | 25 |
| 102 | 71 | 11.5 | 25 |
| 103 | 65 | 46.7 | 23 |
| 104 | 87 | 37.1 | 32 |
| 105 | 77 | 15.2 | 31 |
| 106 | 60 | 44.3 | 30 |
| 107 | 82 | 5.4 | 26 |
| 108 | 58 | 13.4 | 28 |
| 109 | 62 | 7.5 | 25 |
| 110 | 62 | 7.5 | 27 |
| 111 | 50 | 62.2 | 24 |
| 112 | 50 | 62.2 | 23 |
| 113 | 86 | 13.1 | 21 |
| 114 | 86 | 13.1 | 28 |
| 115 | 69 | 45.4 | 21 |

**Supplementary Table S2.** List of antibodies used.

| **Antibody (clone), Host species** | **Antibody dilution** | **Application** | **Antibody source** |
|---|---|---|---|
| Aggrecan, goat | 10 µg/mL | Immunocytochemistry | R&D systems |
| Cadherin-E (24E10), rabbit | 1:200 | Immunohistochemistry | Cell signaling |
| Cadherin-P (A-10), Mouse | 1:100 | Immunohistochemistry | Santa Cruz Biotechnology, INC. |
| CD11c, PE(3.9), Mouse | 5 µl/$10^6$ cells | Flow cytometry | BioLegend |
| CD14 PE(M5E2), Mouse | 5 µl/$10^6$ cells | Flow cytometry | BioLegend |
| CD19 PE(H1B19), Mouse | 5 µl/$10^6$ cells | Flow cytometry | BioLegend |
| CD44 PE(IM7), Mouse | 5 µl/$10^6$ cells | Flow cytometry | BioLegend |
| CD45 PE(H130), Mouse | 5 µl/$10^6$ cells | Flow cytometry | BioLegend |
| CD73 PE(AD2), Mouse | 5 µl/$10^6$ cells | Flow cytometry | BioLegend |
| CD90 PE(5E10), Mouse | 5 µl/$10^6$ cells | Flow cytometry | BioLegend |
| CD90 APC(5E10), Mouse | 5 µl/$10^6$ cells<br>1:400 | Flow cytometry<br>Immunohist/cytochemistry | BD Biosciences |
| CD105 PE(43A3), Mouse | 5 µl/$10^6$ cells | Flow cytometry | BioLegend |
| CD117 PE (A3C6E2), Mouse | 5 µl/$10^6$ cells | Flow cytometry | Miltenyi Biotec |
| Cytokeratin pan (PCK-26), mouse | 1:500 | Immunohistochemistry | Abcam |
| Cytokeratin 3/76 (AE5), mouse | 1:100 | Immunohisto/cytochemistry | Millipore |
| Cytokeratin 12 (EPR17882), rabbit | 1:50 | Immunohistochemistry | Abcam |
| Cytokerain 14 (LL002) Mouse | 1:500 | Immunohisto/cytochemistry | Abcam |
| Cytokeratin 15 (LHK15), mouse | 1:500 | Immunohistochemistry | Abcam |

| | | | |
|---|---|---|---|
| Cytokeratin 15 (EPR1614Y), rabbit | 1:500 | Immunohistochemistry | Abcam |
| Cytokeratin 17/19 (D4G2), rabbit | 1:200 | Immunohisto/cytochemistry | Cell signaling |
| FABP4, goat | 10 µg/mL | Immunocytochemistry | R&D systems |
| HMB-45 (HMB45), mouse | 0.5 µg/ml | Immunocytochemistry | Abcam |
| IgG2a, k, Isotype PE (MOPC-173), mouse | 5 µl/$10^6$ cells | Flow cytometry | Biolegend |
| IgG3, k Isotype FITC (MG3-35), mouse | 5 µl/$10^6$ cells | Flow cytometry | Biolegend |
| IgG2a, k Isotype APC (eBM2a), mouse | 5 µl/$10^6$ cells | Flow cytometry | Invitrogen |
| Ki-67 (EPR3610), rabbit | 1:500 | Immunohistochemistry | Abcam |
| Osteocalcin, mouse | 10 µg/mL | Immunocytochemistry | R&D systems |
| Melan A, (EPR20380), rabbit | 1:1000 | Immunohisto/cytochemistry | Abcam |
| Melan A, (788), mouse | 0.5 µg/mL | Immunohisto/cytochemistry | Novusbio |
| p63 (Y289), rabbit | 1:200 | Immunohistochemistry | Abcam |
| Sox10 (EPR4007) | 1:500 | Immunocytochemistry | Abcam |
| TRP1 (EPR21960) | 1:1000 | Immunocytochemistry | Abcam |
| Vimentin, (D21H3), rabbit | 1:500 | Immunohistochemistry | Cell Signaling |
| Vimentin (280618), rat | 8 µg/mL | Immunohisto/cytochemistry | R&D systems |
